# Supplementary figures and images for: Sex determination gene transformer regulates the male-female difference in Drosophila fat storage via the adipokinetic hormone pathway
Source: eLife. 2021 Oct 21;10:e72350. doi: 10.7554/eLife.72350 (PMC8594944; doi:10.7554/eLife.72350)

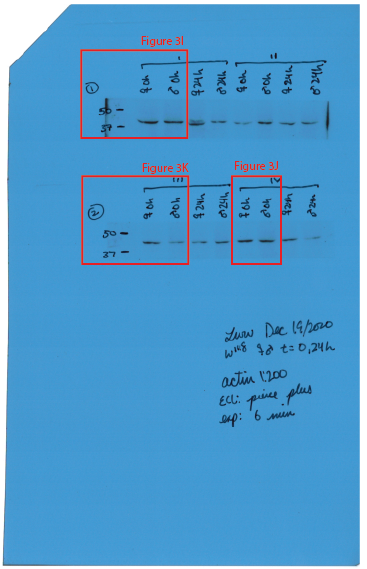

Supplement: Figure 3—source data 3. [file elife-72350-fig3-data3.zip › Figure 3 - Source Data 3 (WB)/Figure 3 - actin western blot source data.png]

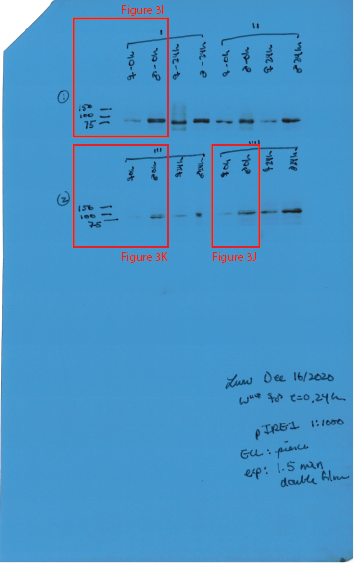

Supplement: Figure 3—source data 3. [file elife-72350-fig3-data3.zip › Figure 3 - Source Data 3 (WB)/Figure 3 - pIre1 western blot source data.png]

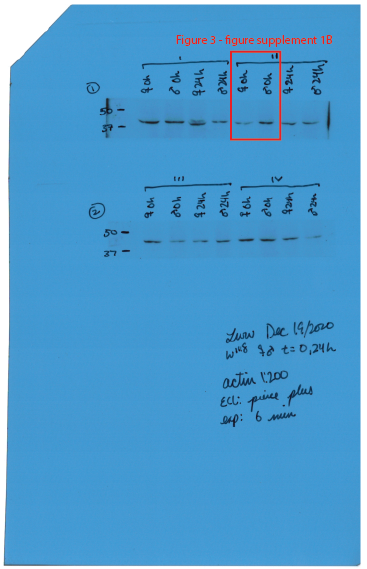

Supplement: Figure 3—figure supplement 1—source data 1. [file elife-72350-fig3-figsupp1-data1.zip › Figure 3 - figure supplement 1 - Source Data 1 (WB)/Figure 3 - figure supplement 1 - actin western blot source data.png]

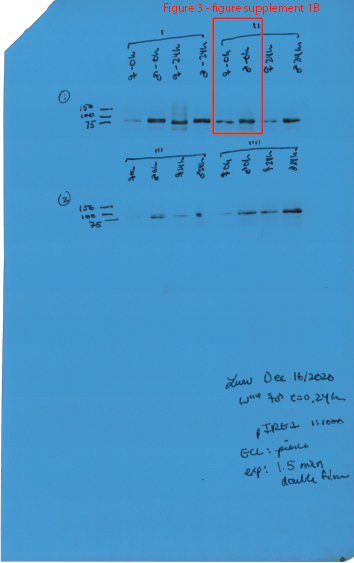

Supplement: Figure 3—figure supplement 1—source data 1. [file elife-72350-fig3-figsupp1-data1.zip › Figure 3 - figure supplement 1 - Source Data 1 (WB)/Figure 3 - figure supplement 1 - pIre1 western blot source data.png]

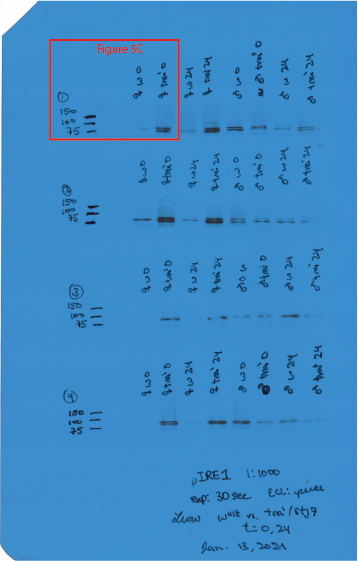

Supplement: Figure 5—source data 1. [file elife-72350-fig5-data1.zip › Figure 5 - Source Data 1 (WB)/Figure 5 - pIre1 western blot source data pt2.png]

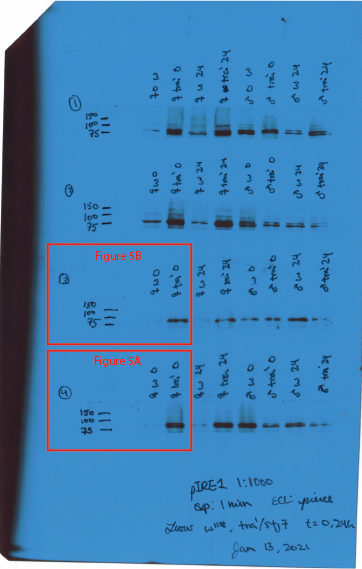

Supplement: Figure 5—source data 1. [file elife-72350-fig5-data1.zip › Figure 5 - Source Data 1 (WB)/Figure 5 - pIre1 western blot source data pt1.png]

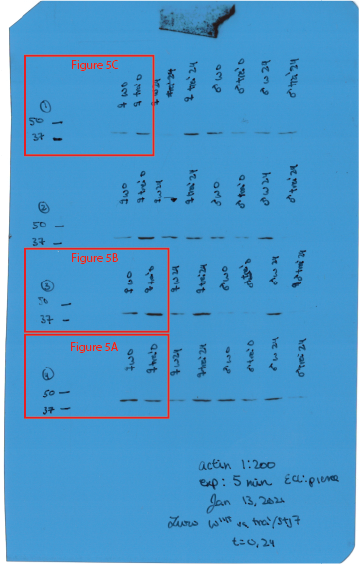

Supplement: Figure 5—source data 1. [file elife-72350-fig5-data1.zip › Figure 5 - Source Data 1 (WB)/Figure 5 - actin western blot source data.png]

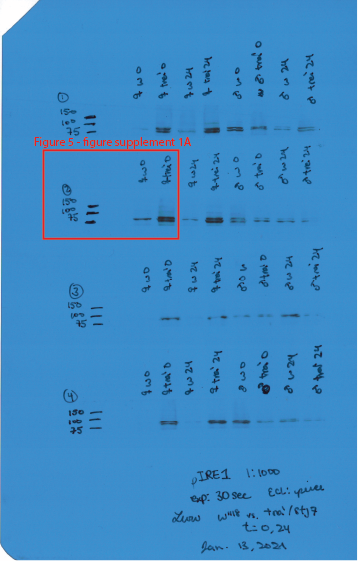

Supplement: Figure 5—figure supplement 1—source data 1. [file elife-72350-fig5-figsupp1-data1.zip › Figure 5 - figure supplement 1 - Source Data 1 (WB)/Figure 5 - figure supplement 1 - pIre1 western blot source data.png]

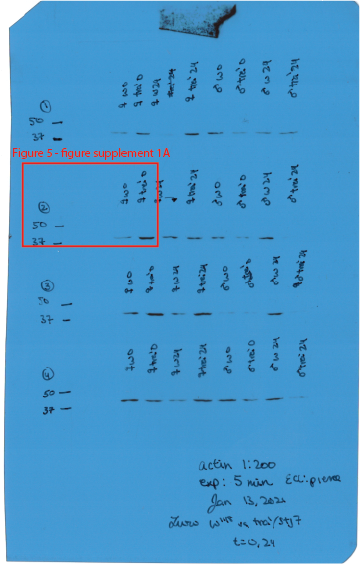

Supplement: Figure 5—figure supplement 1—source data 1. [file elife-72350-fig5-figsupp1-data1.zip › Figure 5 - figure supplement 1 - Source Data 1 (WB)/Figure 5 - figure supplement 1 - actin western blot source data.png]
